# A CsoR family transcriptional regulator, TTHA1953, controls the sulfur oxidation pathway in *Thermus thermophilus* HB8

**DOI:** 10.1016/j.jbc.2023.104759

**Published:** 2023-04-26

**Authors:** John K. Barrows, Michael W. Van Dyke

**Affiliations:** Department of Chemistry and Biochemistry, Kennesaw State University, Kennesaw, Georgia, USA

**Keywords:** bacterial transcription, DNA-binding protein, transcription factor, sulfur, extreme thermophile

## Abstract

Transcription regulation is a critical means by which microorganisms sense and adapt to their environments. Bacteria contain a wide range of highly conserved families of transcription factors that have evolved to regulate diverse sets of genes. It is increasingly apparent that structural similarities between transcription factors do not always equate to analogous transcription regulatory networks. For example, transcription factors within the copper-sensing operon repressor (CsoR)–resistance to cobalt and nickel repressor family have been found to repress a wide range of gene targets, including various metal efflux genes, as well as genes involved in sulfide and formaldehyde detoxification machinery. In this study, we identify the preferred DNA-binding sequence for the CsoR-like protein, TTHA1953, from the model extremophile *Thermus thermophilus* HB8 using the iterative selection approach, restriction endonuclease, protection, selection, and amplification. By mapping significant DNA motifs to the *T. thermophilus* HB8 genome, we identify potentially regulated genes that we validate with *in vitro* and *in vivo* methodologies. We establish TTHA1953 as a master regulator of the sulfur oxidation pathway, providing the first link between CsoR-like proteins and *Sox* regulation.

The copper-sensing operon repressor (CsoR) and resistance to cobalt and nickel repressor (RcnR) family of transcriptional regulators is widely abundant in bacteria (1). CsoR–RcnR homologs are small (∼10 kDa) proteins containing several alpha helices that adopt multimeric complexes to bind DNA. Traditional CsoR–RcnR members repress metal efflux genes in the absence of a regulatory metal cofactor (2, 3). Coordination of a metal effector promotes a conformational change in the CsoR–RcnR complex, allowing derepression of the metal efflux genes. Common metal effectors are copper, nickel, and cobalt; although several transcription regulators with unique effector molecules have been added to the CsoR–RcnR family based on structural similarities, including the formaldehyde-responsive regulator (4) and the CsoR-like sulfur transferase repressor (CstR) (5), which senses reactive sulfur species. As such, it is common for bacteria to encode for more than one CsoR–RcnR family member. Most CsoR–RcnR homologs bind pseudo–palindromic DNA sequences containing a continuous stretch of ≥4 guanines or cytosines (6). This sequence specificity is highlighted by the *Mycobacterium tuberculosis* CsoR DNA-binding sequence (5′-GTAGCCCACCCCCAGTGGGGTGGATAC-3′), which overlaps the −10 and −35 elements of *cso* (copper-sensitive operon) (3).

Sulfur-oxidizing bacteria (SOB) rely on the metabolism of inorganic sulfur compounds for the energy needed to survive and reproduce. A common substrate for SOB is thiosulfate, which can be oxidized to sulfate using a variety of different pathways (7, 8, 9). One of the more common ways SOB oxidize thiosulfate to sulfate is through the sulfur oxidation (Sox) pathway, which includes a multitude of periplasmic enzymes that catalyze the transfer and removal of electrons from thiosulfate (8). The core components of the Sox pathway include the heterodimer proteins SoxXA, SoxYZ, and SoxCD as well as SoxB (10). When presented with thiosulfate, SoxXA, a heterodimeric c-type cytochrome, conjugates thiosulfate to a cysteine residue on SoxYZ (11). SoxB then removes a sulfonate group from thiosulfate-conjugated SoxYZ (12). At this point, SoxYZ can accept another thiosulfate conjugate (13), or SoxCD can oxidize the terminal sulfane group to sulfonate (14). The remaining sulfonate group can then be removed by SoxB, and the process restarted. Genes involved in the Sox pathway are often cotranscribed; however, the mechanisms behind *Sox* regulation are not well established.

In this study, we identify a consensus DNA-binding sequence for the CsoR-like protein, TTHA1953, from the model extremophile, *Thermus thermophilus* HB8. *T. thermophilus* HB8 encodes for two CsoR-like proteins, TTHA1719 and TTHA1953. TTHA1719 was previously characterized as a *bona fide* CsoR that represses the *cso* (containing *copZ*, *csoR*, and *copA*) in its apo form (15). Notably, we show TTHA1719 and TTHA1953 recognize unique DNA sequences. We then characterize the transcription regulatory network for TTHA1953, which includes the core enzymes of the Sox pathway. Ablation of *TTHA1953* expression resulted in reduced growth efficiency for *T. thermophilus* HB8 that was partially rescued with thiosulfate supplementation. Collectively, these results provide a molecular link between CsoR-like transcription regulators and the Sox pathway in SOB.

## Results

### Identification of high-affinity TTHA1953 DNA-binding sequences by restriction endonuclease, protection, selection, and amplification

To investigate the biological function of a previously uncharacterized CsoR-like protein from *T. thermophilus* HB8, encoded by *TTHA1953*, we first performed a position-specific iterative BLAST against the Protein Data Bank database (16). While the distance tree of the BLAST results showed a variety of CsoR–RcnR homologs, the closest homology to TTHA1953 was *bona fide* CsoR proteins (Fig. S1). Since *T. thermophilus* HB8 contains an experimentally validated CsoR (encoded by *TTHA1719*), it is unlikely that TTHA1953 is a functionally redundant CsoR. Therefore, we sought an alternative method to elucidate the molecular function of TTHA1953.

**Figure 1:**
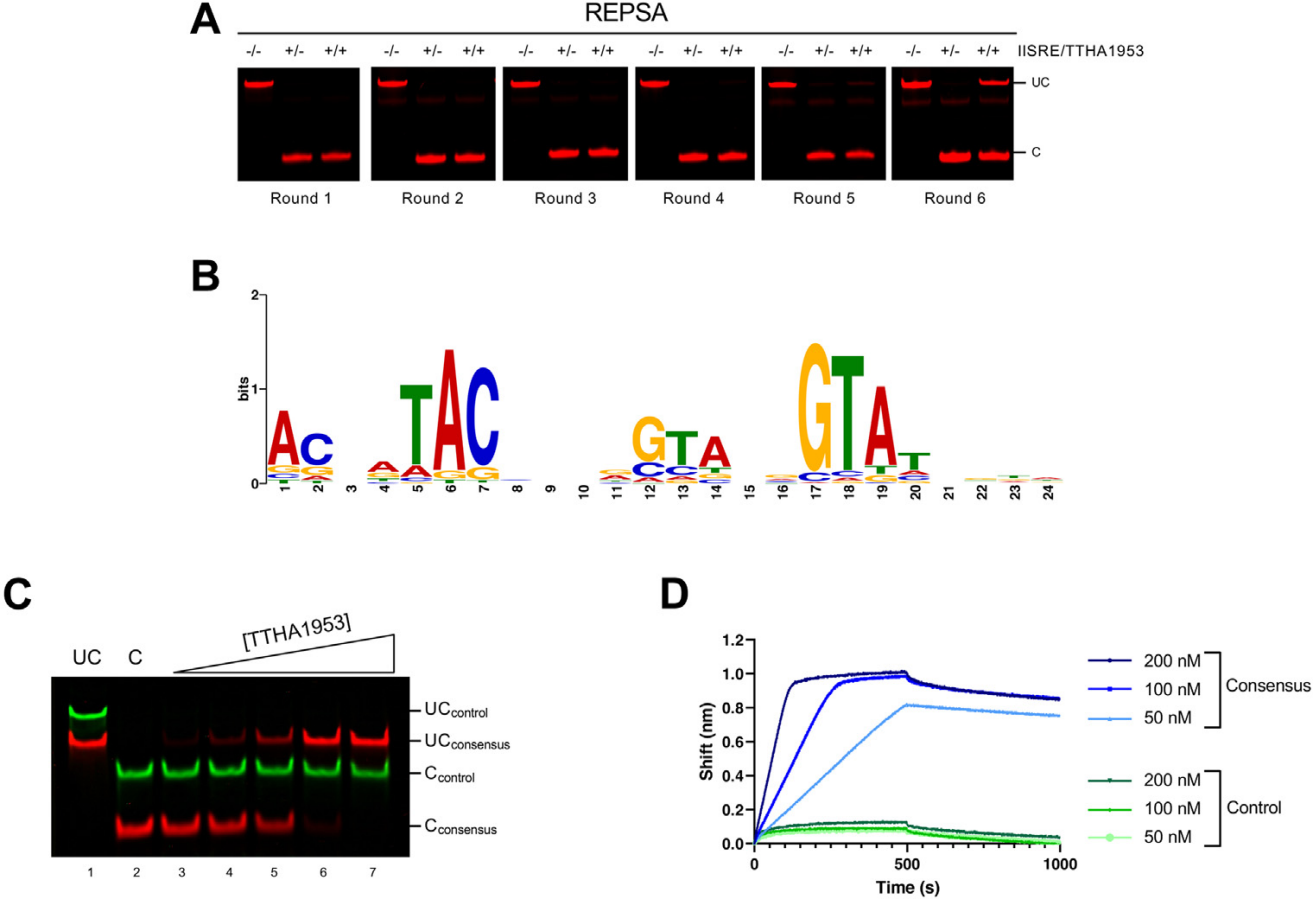
**Preferred DNA-binding sequence for TTHA1953 identified by REPSA.***A*, REPSA was performed using a 73-bp selection template containing an internal 24-bp region of random nucleotides. DNAs for each round were incubated for 20 min with buffer or 60 nM TTHA1953 and then challenged with a type II restriction endonuclease (IISRE). FokI and BpmI were used as IISREs, and their use alternated between consecutive rounds (rounds 1, 3, and 5 used FokI; rounds 2, 4, and 6 used BpmI). Uncut (UC) and cut (C) DNAs are labeled. *B*, DNAs from round 6 of REPSA were analyzed by high-throughput sequencing. Sequencing reads were then input into MEME software, and the position weight matrix for the most significant motif is presented. *C*, DNAs containing our consensus TTHA1953 DNA-binding sequence (5′ IRDye700-labeled; *red*) and control DNAs with no sequence homology (5′IRDye800-labeled; *green*) were incubated with 7.5, 15, 30, 60, or 120 nM TTHA1953 for 20 min and then treated with FokI. UC and C DNAs are labeled. *D*, the binding of TTHA1953 to DNA probes containing our consensus TTHA1953 DNA-binding sequence (*blue*) or a control sequence with no homology (*green*) was analyzed by biolayer interferometry. The association (time < 500 s) and dissociation (time > 500 s) steps are presented. MEME, Multiple Em for Motif Elicitation; REPSA, restriction endonuclease, protection, selection, and amplification.

To do so, we performed restriction endonuclease, protection, selection, and amplification (REPSA) using recombinant TTHA1953. REPSA is an established iterative selection technique used to identify the preferred DNA-binding sequences of a protein of interest (17). In the initial round of REPSA, a DNA pool containing a defined region of randomized nucleotides is incubated with a transcription factor of interest and challenged with a type IIS restriction endonuclease (IISRE). Transcription factor–bound DNAs are protected from IISRE cleavage, amplified by PCR, and then used as inputs to the next round of REPSA. This process is repeated until a protected DNA species is observed by gel electrophoresis. Using a selection template library containing a 24 base pair (bp) region of random nucleotides, we conducted six rounds of REPSA with recombinant TTHA1953 (Fig. 1*A*). We observed that a minor protected DNA population in round 5 and a more predominant protected DNA population in round 6. The emergence of a protected DNA species in later rounds of REPSA is indicative of a successful selection of high-affinity TTTHA1953 DNA-binding sequences. To ascertain the nucleotide sequence of the protected DNAs, round 6 DNAs were amplified with barcodes compatible for Illumina-based technologies, and high-throughput DNA sequencing was performed using an iSeq100 system. This library yielded 418,806 sequences with 80.19% Q30. Resulting DNA sequences were trimmed and inputted into Multiple Em for Motif Elicitation (MEME; https://meme-suite.org/meme/tools/meme; accessed August 26, 2022) software to identify significant DNA motifs (18, 19). The position weight matrix of the most highly significant motif is presented in Figure 1*B*. The depicted motif was found in ∼40% of the input sequences and had an E-value of 1.1E-965. The identified motif spans the entire length of the 24 bp random sequence, while seemingly containing four random nucleotides in spaces 21 to 24. This phenomenon will occur when the ligand of interest binds one or more base pairs within the defined flanking region of the selection template, thus preferentially biasing the motif to one side of the random cassette. If the presented motif in Figure 1*B* were to be a palindromic sequence, one would expect a thymine at position 0 (since an adenine is found at position 19), and indeed, there is a thymine at the 0 position in our selection template. Therefore, we identified the consensus DNA-binding sequence for TTHA1953 as TACNNTACNNNNGTANNGTA. This sequence can be described as a 20 bp palindrome comprised of two 8 bp inverted repeat sequences separated by four nucleotides. Each 8 bp inverted repeat contains two three-nucleotide repeats of either TAC or GTA. The presence of a single 5′-TAC/GTA-3′ inverted repeat has been previously identified in several CsoR-binding sequences; however, our identified motif lacks a continuous stretch of G/C bases commonly found in other CsoR–RcnR-binding sequences (20).

### Validation and mutagenesis of consensus TTHA1953 DNA-binding sequence

To confirm TTHA1953 binding to our REPSA-identified consensus motif, we developed oligos containing the following sequence: TACTATACCTTGGTATGGTA; where highly conserved nucleotides from our consensus motif are underlined. We initially tested the ability of TTHA1953 to bind this DNA sequence by restriction endonuclease protection assay (REPA). Similar to REPSA but excluding the PCR steps, REPAs analyze transcription factor binding to DNA sequences by challenging protein–DNA solutions with a IISRE. As seen in Figure 1*C*, increasing concentrations of TTHA1953 resulted in nearly 100% IISRE cleavage protection of our consensus sequence probe (shown in *red*), whereas a control probe containing no sequence homology (shown in *green*) was susceptible to IISRE cleavage at all concentrations of TTHA1953 tested. To analyze binding dynamics in real time, we assayed TTHA1953 binding to our consensus sequence and a control sequence by biolayer interferometry (BLI) (Fig. 1*D*). When our consensus probe was incubated with various concentrations of TTHA1953 (time <500 s), we observed shifts consistent with TTHA1953 binding. When these complexes were subjected to extensive dilution (time >500 s), the changes in wavelength decreased only slightly, indicative of high-affinity TTHA1953–DNA complexes. Conversely, the addition of TTHA1953 to a control probe with no sequence homology to our consensus motif resulted in little to no shift in wavelength. Collectively, these data show that TTHA1953 specifically associates with the consensus sequence we identified through REPSA.

To formally test the sequence specificity of TTHA1953 to our consensus sequence, we developed an array of mutagenic oligos and assayed TTHA1953 binding by BLI (Table 1). Mutating single nucleotides found within one inverted repeat region reduced the dissociation constant, *K*_*d*_, by ∼2- to 4.5-fold compared with the consensus sequence, with the largest reduction occurring by mutating the position immediately next to the 4 bp region separating the inverted repeats. Adding or removing a nucleotide in the region between inverted repeats reduced the *K*_*d*_ by ∼10- or 15-fold, respectively, and mutating two sets of the three nucleotide repeats (TAC/GTA) completely abolished detectable binding. Collectively, these results show that TTHA1953 can tolerate single point mutations in its consensus sequence; however, the spacing between the two inverted repeats is important for high-affinity binding.

**Table 1:** Mutational analysis of TTHA1953 consensus DNA-binding sequence

Mutations in the consensus binding sequence (WT) are indicated in *bold* or *red font*.

Abbreviation: N/A, not available.

^*a*^ Kinetic values (*k*_on_, *k*_off_, and *K*_*d*_) were determined using a nonlinear regression, *Association then Dissociation* model in GraphPad Prism 9.

The consensus motif we identified through REPSA presented a noticeably different DNA sequence compared with the binding sequence of the other CsoR-like homolog in *T. thermophilus* HB8, TTHA1719 (referred to hereafter as *Tt*CsoR) (15). The *Tt*CsoR DNA-binding sequence (CCCCACCCCACCTGGGGTGGGG) is pseudopalindromic and G/C rich, consistent with previous CsoR–RcnR-binding sequences. To test whether the two *T. thermophilus* HB8 CsoR-like homologs had similar DNA-binding sequence specificities, we analyzed binding of both CsoR family proteins to the established *Tt*CsoR DNA-binding sequence and to our TTHA1953 consensus DNA-binding sequence (Fig. S2, *A* and *B*). Notably, we observed little to no binding of TTHA1953 to the *Tt*CsoR-binding sequence as well as little to no binding of *Tt*CsoR to our TTHA1953 consensus sequence. This result suggests that the two *T. thermophilus* HB8 CsoR-like proteins have unique transcription regulatory networks.

### TTHA1953 preferentially binds DNA when fully reduced

**Figure 2:**
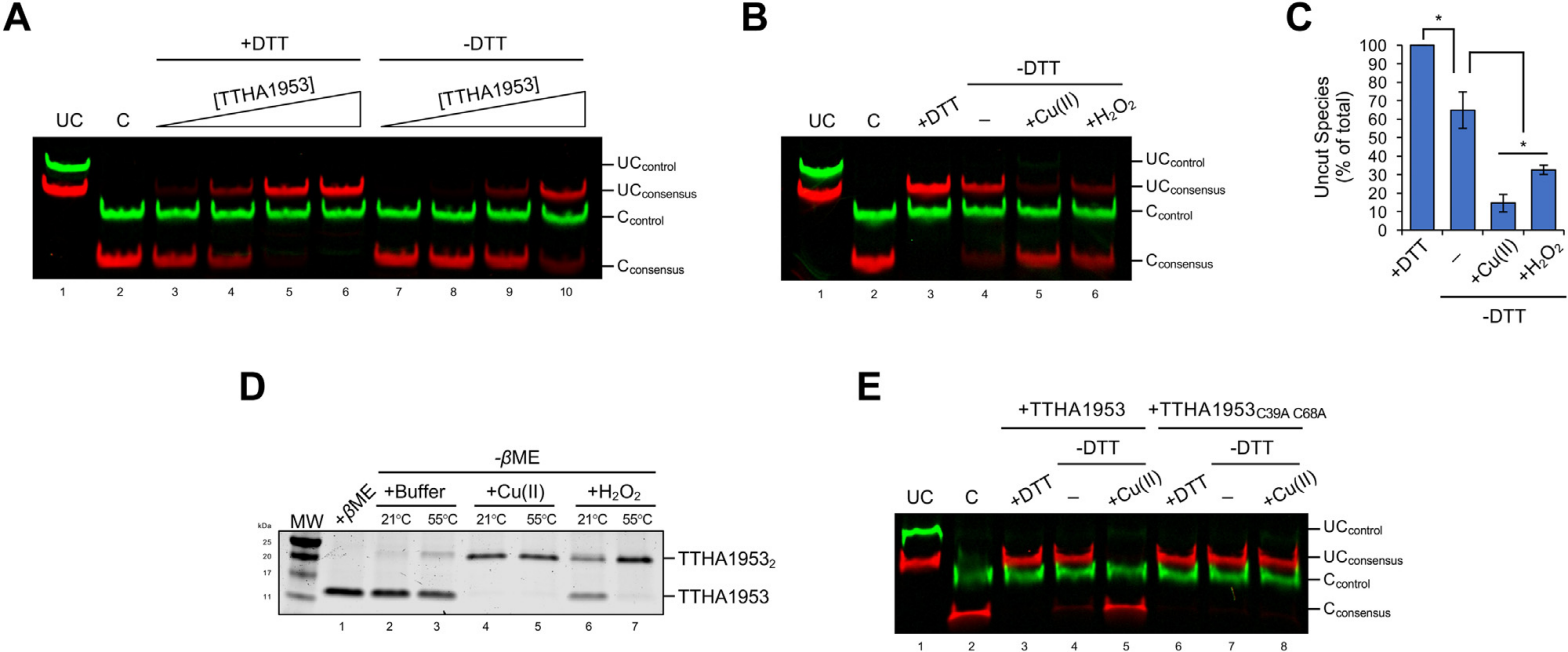
**TTHA1953 preferentially binds DNA under reduced conditions.***A*, DNAs containing our consensus TTHA1953 DNA-binding sequence (5′ IRDye700-labeled; *red*) and control DNAs with no sequence homology (5′IRDye800-labeled; *green*) were incubated with 15, 30, 60, or 120 nM TTHA1953 in the presence or the absence of 1 mM DTT for 20 min and then treated with FokI. Uncut (UC) and cut (C) DNAs are labeled. *B*, DNAs containing our consensus TTHA1953 DNA-binding sequence (5′ IRDye700-labeled; *red*) and control DNAs with no sequence homology (5′IRDye800-labeled; *green*) were incubated with 120 nM TTHA1953 in the presence or the absence of 1 mM DTT. Where indicated, reactions were supplemented with 100 μM CuCl_2_ or 100 μM H_2_O_2_. Reactions were incubated for 20 min and then treated with FokI. UC and C DNAs are labeled. *C*, relative amounts of UC consensus DNAs from (*B*) were quantified. Error bars represent ±1 standard deviation between two independent experiments. Student’s two-tailed *t* test; ∗*p* < 0.05. *D*, 2 μg of recombinant TTHA1953 was treated with a buffer control or 10 mM CuCl_2_ or H_2_O_2_ for 20 min at the indicated temperature. Each sample was diluted with 1× Laemmli buffer in the presence or the absence of 350 mM β-mercaptoethanol (βME) and then resolved by SDS-PAGE. Proteins were imaged by Coomassie blue staining. *E*, DNAs containing our consensus TTHA1953 DNA-binding sequence (5′ IRDye700-labeled; *red*) and control DNAs with no sequence homology (5′IRDye800-labeled; *green*) were incubated with 120 nM TTHA1953 or TTHA1953_C39A C68A_ in the presence or the absence of 1 mM DTT. Where indicated, reactions were supplemented with 100 μM CuCl_2_. Reactions were incubated for 20 min and then treated with FokI. UC and C DNAs are labeled. MW, molecular weight ladder.

Previously characterized CsoR-like proteins can bind DNA as a dimer of tetramers (20, 21). Consistent with these findings, we found TTHA1953 likely binds its consensus sequence DNA as a homo-octomer complex (apparent weight of TTHA1953 DNA-binding complex: ∼85 kDa; estimated homo-octomer weight: 83 kDa) using an adaptation of a Ferguson plot (22) (Fig. S3). Traditional CsoR and RcnR proteins coordinate a metal ion to release from DNA, allowing derepression of metal efflux genes (1). Common regulatory metals for this family are copper, nickel and cobalt; however, none of these metals abolished TTHA1953 DNA binding under reduced conditions (Fig. S4). Conversely, we did observe a reduction in TTHA1953 DNA-binding affinity in the absence of a reducing agent (Fig. 2*A*), suggesting the two cysteine residues present in TTHA1953 play a role in regulating DNA binding (Fig. S5) (23). To test whether thiol reactivity affects DNA binding of TTHA1953 *in vitro*, we performed a REPA under nonreducing conditions in the presence of two molecules known to react with thiol groups, Cu(II) and H_2_O_2_ (24, 25, 26). Incubation with either molecule resulted in a significant reduction of TTHA1953 binding affinity under nonreducing conditions (Fig. 2, *B* and *C*). We then used nonreducing SDS-PAGE to analyze the oligomer state of TTHA1953 incubated in either buffer, Cu(II) or H_2_O_2_ (Fig. 2*D*). When TTHA1953 was incubated with Cu(II) or H_2_O_2_, we observed a substantial increase in a larger molecular weight species, approximately twice the molecular weight of monomeric TTHA1953. This result suggests that the addition of either Cu(II) or H_2_O_2_ promotes disulfide-linked TTHA1953 dimers *in vitro*. To further address the role of disulfide linkages in the DNA-binding ability of TTHA1953, we created a mutant construct of TTHA1953 that contained alanine substitutions for both cysteine residues (referred to as TTHA1953_C39A C68A_). We then analyzed binding of TTHA1953_C39A C68A_ to our consensus DNA sequence by REPA (Fig. 2*E*). Notably, unlike wildtype TTHA1953, the ability of TTHA1953_C39A C68A_ to protect against IISRE digestion was not substantially altered in nonreducing reactions or nonreducing reactions containing Cu(II). This result shows that the DNA-binding affinity of TTHA1953 remains unchanged when the protein is unable to form disulfide linkages; and collectively, these data demonstrate that covalent coupling of thiol groups between TTHA1953 monomers reduces DNA-binding affinity *in vitro*.

### *In vitro* validation of genomic DNA-binding sequences

**Figure 3:**
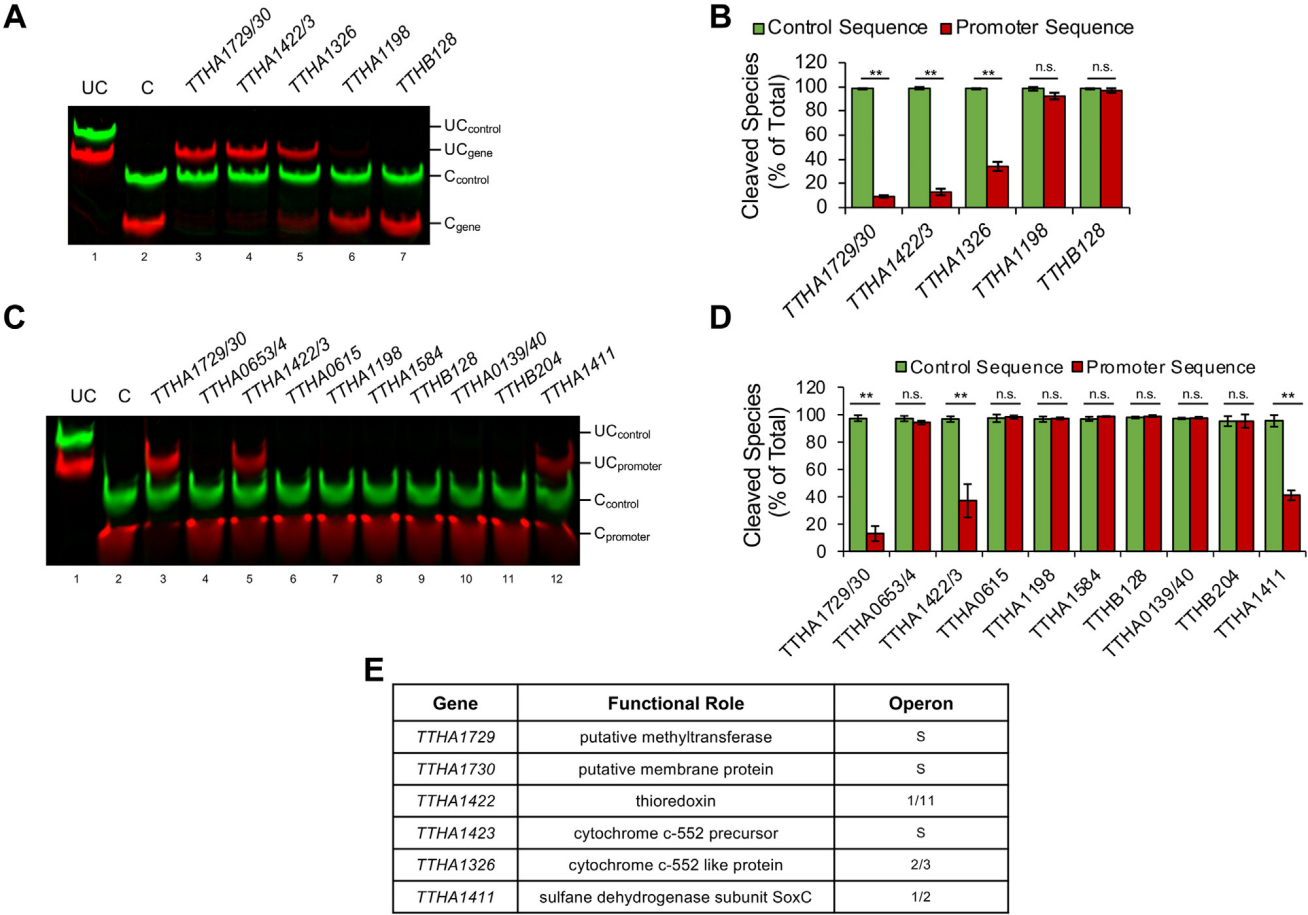
***In vitro* validation of genomic TTHA1953-binding sequences.***A*, DNAs containing 20-nt sequences from the FIMO output in Fig. S6*B* (5′ IRDye700-labeled; *red*) and control DNAs with no sequence homology (5′IRDye800-labeled; *green*) were incubated with 100 nM TTHA1953 for 20 min and then treated with FokI. Uncut (UC) and cut (C) DNAs are labeled. *B*, the intensity of cleaved DNAs from (*A*) was graphed for the indicated sequences. Error bars represent ±1 standard deviation between three independent experiments. Student’s two-tailed *t* test; n.s., not significant; ∗∗*p* < 0.005. *C*, DNAs containing 21-nt sequences from the FIMO output in Fig. S6*D* (5′ IRDye700-labeled; *red*) and control DNAs with no sequence homology (5′IRDye800-labeled; *green*) were incubated with 100 nM TTHA1953 for 20 min and then treated with FokI. UC and C DNAs are labeled. *D*, the intensity of cleaved DNAs from (*C*) was graphed for the indicated sequences. Error bars represent ±1 standard deviation between three independent experiments. Student’s two-tailed *t* test; n.s., not significant; ∗∗*p* < 0.005. *E*, representation of genes found in close proximity (<200 nts) of genomic sequences that exhibited significant cleavage protection in (*A*–*D*). Functional roles were predicted by GenBank. Operon, gene position within postulated operon; S, single transcription unit. FIMO, Find Individual Motif Occurrences.

To discover potential genes in the TTHA1953 regulatory network, we uploaded two slight variations of the consensus motif identified by MEME into Find Individual Motif Occurrences (FIMO; https://meme-suite.org/meme/tools/fimo; accessed August 26, 2022) (27) (motifs either lacked or included position 20 in Fig. 1*B*). The output of this analysis depicts sequences within the *T. thermophilus* HB8 genome that significantly match the inputted position weight matrix (Fig. S6). From these FIMO outputs, we created oligos containing significant DNA sequences (*p* value < 3.5E-6) found within promoter regions (defined as within −200/+20 bp of a transcription start site). As expected, several sequences were found in both FIMO outputs with similar significance. We assayed TTHA1953 binding to these genomic sequences by REPA (Fig. 3, *A* and *C*). We found four genomic sequences within gene promoters that exhibited significant cleavage protection in the presence of TTHA1953 compared with a control sequence with no homology (Fig. 3, *B* and *D*). The sequences were found upstream of the following genes: *TTHA1729*, *TTHA1730*, *TTHA1422*, *TTHA1423*, *TTHA1326*, and *TTHA1411*. Therefore, these results implicate TTHA1953 in the regulation of six transcription units (four TTHA1953-specific promoter-binding sequences, two of which contain divergent genes). The proteins encoded by these genes varied in molecular function, including a methyltransferase, an uncharacterized membrane protein, a thioredoxin, a sulfane dehydrogenase, and two cytochrome *c*-552-related proteins (Fig. 3*E*). Notably, previous studies identified the *c*-type cytochrome encoded by *TTHA1326* as a player in sulfite oxidation (28), and the genes immediately downstream of *TTHA1422*, including *TTHA1411*, contain highly conserved members of a Sox system. The biological role of these transcription units suggests that TTHA1953 regulates genes involved in sulfur metabolism, similar to CstR (5).

### TTHA1953 controls the Sox pathway *in vivo*

**Figure 4:**
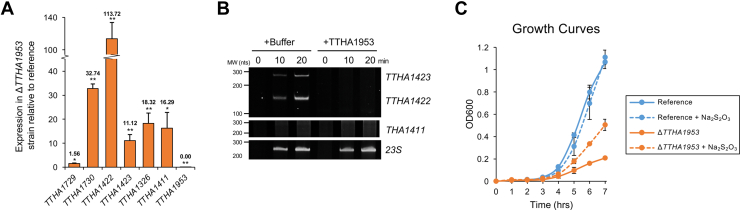
**TTHA1953 is a master regulator of the sulfur oxidation pathway.***A*, RNA was isolated from reference and *TTHA1953*-disrupted (Δ*TTHA1953*) *Thermus thermophilus* HB8, and gene expression was quantified by RT–quantitative PCR. Each gene was normalized to endogenous *TTHA0185* expression, and Δ*TTHA1953* expression values relative to the reference strain are shown. Error bars represent ±1 standard deviation between three independent experiments. Student’s two-tailed *t* test; ∗*p* < 0.05; ∗∗*p* < 0.005. *B*, DNA templates including the proximal promoters and downstream regions of the indicated genes were incubated with *T. thermophilus* HB8 RNA polymerase holoenzyme and sigma-70 in the presence or the absence of TTHA1953. RNA was analyzed by 5% TBE–urea PAGE and visualized with SYBR Gold staining. *C*, reference and *TTHA1953*-disrupted *T. thermophilus* HB8 strains were grown in TT media (see Experimental procedures section) in the presence or the absence of 10 mM sodium thiosulfate; n = 2.

To analyze the TTHA1953 transcription regulatory network *in vivo*, we created *TTHA1953*-disrupted strains of *T. thermophilus* HB8 in which *TTHA1953* was replaced with a kanamycin-resistance gene (29). Kanamycin-resistant colonies were isolated, and disruption of *TTHA1953* was validated by genomic PCR (Fig. S7). RNA from reference and Δ*TTHA1953 T. thermophilus* HB8 cultures was isolated, and gene expression was analyzed by RT–quantitative PCR (qPCR) (Fig. 4*A*). As expected, the expression of *TTHA1953* was severely diminished compared with the reference strain. Notably, five of the six genes we identified as containing a TTHA1953-binding site in their promoter exhibited >10-fold increase in expression in the absence of *TTHA1953*, with the one exception being *TTHA1729*, which had a significant, yet subtle, increase in expression (∼1.5-fold). The gene with the highest fold change in expression in the Δ*TTHA1953* strain relative to the reference strain was *TTHA1422*. As mentioned previously, the genes immediately downstream of *TTHA1422* encode for a Sox system: a periplasmic multiprotein complex that oxidizes thiosulfate to sulfate in SOB. In the absence of *TTHA1953*, we observed significant increases in the expression of all genes from *TTHA1421* to *TTHA1410* (Table 2). This range of genes contains homologous Sox proteins including SoxA (*TTHA1415*), SoxB (*TTHA1417*), SoxC (*TTHA1411*), SoxD (*TTHA1410*), SoxX (*TTHA1416*), SoxY (*TTHA1421*), and SoxZ (*TTHA1420*). Using bacterial promoter and terminator identification software, we found putative promoter sequences upstream of both *TTHA1422* and *TTHA1411* as well as putative terminator sequences after *TTHA1412* and *TTHA1410* (Fig. S8). This suggests that Sox genes in *T. thermophilus* HB8 are predominantly controlled by two operons, and both operons harbor TTHA1953 DNA-binding sequences.

**Table 2:** Expression of genes downstream of *TTHA1422* in Δ*TTHA1953* strains

Gene	Functional role	Fold increase	Standard deviation	*p*
*TTHA1422*	Thioredoxin (SoxW)	113	20.0	6.2E-4
*TTHA1421*	Sulfur oxidation protein SoxY	33.4	6.10	7.8E-4
*TTHA1420*	Sulfur oxidation protein SoxZ	77.6	8.27	8.9E-5
*TTHA1419*	l-cysteine S-thiosulfotransferase	24.7	4.59	8.8E-4
*TTHA1418*	l-cysteine S-thiosulfotransferase	32.9	8.11	0.0024
*TTHA1417*	Sulfur oxidation protein SoxB	8.17	1.44	0.0010
*TTHA1416*	Sulfur oxidation protein SoxX	16.0	1.50	7.6E-5
*TTHA1415*	Sulfur oxidation protein SoxA	8.87	1.40	6.6E-4
*TTHA1414*	Putative sulfurtransferase	2.86	0.78	0.021
*TTHA1413*	Hypothetical protein	5.46	2.31	0.031
*TTHA1412*	Sulfide dehydrogenase flavocytochrome C	22.7	9.82	0.019
*TTHA1411*	Sulfane dehydrogenase subunit SoxC	16.3	6.53	0.016
*TTHA1410*	S-disulfanyl-l-cysteine oxidoreductase SoxD	20.8	9.17	0.020
*TTHA1409*	Cytochrome *c*-type biogenesis protein CcdA	1.15	0.33	0.47

*Functional role*, predicted function of encoded protein determined by GenBank; *Fold increase*, relative to reference strain; *p* value, Student’s two-tailed *t* test, n = 3.

To further connect TTHA1953 to *Sox* regulation, we performed an *in vitro* transcription assay using a template containing the divergent promoter for *TTHA1423* and *TTHA1422*. In the presence of TTHA1953, transcription in either direction was completely abrogated (Fig. 4*B*), consistent with the significant increase in expression observed in Δ*TTHA1953* strains. The addition of TTHA1953 to a template containing the *23S* promoter sequence had no effect on transcription levels (Fig. 4*B*), showing that the transcription suppression observed on the *TTHA1422/3* template was dependent on DNA sequence. We observed little to no RNA product when using a template containing the promoter region upstream of *TTHA1411* regardless of TTHA1953 addition (Fig. 4*B*), suggesting the *TTHA1411* promoter is much weaker than the *TTHA1422* promoter or needs additional cofactors to activate transcription to the activity observed by the *TTHA1422* promoter.

SOB often rely on the Sox pathway for energy and electron transfer. Interestingly, in the absence of *TTHA1953*, we noticed a severe delay in growth when cultured on solid media (Fig. S9) or in liquid media (Fig. 4*C*) suggesting TTHA1953 is critical for optimal *T. thermophilus* HB8 growth. Consistent with dysregulation of the Sox pathway, the addition of 10 mM thiosulfate to Δ*TTHA1953* cultures resulted in a partial rescue of the observed growth defect (Fig. 4*C*, compare *orange lines*). The addition of similar amounts of thiosulfate had little to no effect on the growth of the reference strain (Fig. 4*C*, compare *blue lines*). Together, these data suggest that *T. thermophilus* HB8 uses thiosulfate as a primary energy source and cannot grow efficiently when sulfur metabolism genes are unregulated. Together, these data, along with the expression data from Δ*TTHA1953* strains, formally implicate the CsoR-like protein, TTHA1953, as a major regulator of the Sox pathway in *T. thermophilus* HB8.

## Discussion

In this study, we provide a biochemical characterization of the CsoR-like protein, TTHA1953, from *T. thermophilus* HB8. Using REPSA, we identified a highly significant DNA-binding motif. Then, we mapped this motif to the *T. thermophilus* HB8 genome and identified TTHA1953 regulatory genes that were validated using *in vitro* and *in vivo* methodologies. By doing so, we formally implicate TTHA1953 in the transcriptional repression of the Sox pathway.

### DNA-binding and transcription regulatory network of TTHA1953

Our discovered DNA-binding motif for TTHA1953 (Fig. 1*B*) presents a novel sequence specificity compared with previous CsoR–RcnR family members. Typically, DNA-binding sequences of CsoR–RcnR proteins contain a continuous stretch of four or more guanine or cytosine bases (6, 20), which is absent in our DNA-binding motif. It is worth noting that continuous stretches of guanines or cytosines may not be adequately represented in our selection template or may not be amenable to efficient PCR amplification. Regardless, our wildtype consensus sequence does not contain any more than two repetitive guanines/cytosines, yet TTHA1953 binds this sequence with high affinity (Fig. 1, *C* and *D*). It is also noteworthy that all four, experimentally validated, TTHA1953 genomic binding sequences contain central G/C-rich regions; however, none of these sequences contain a continuous stretch of guanine or cytosine bases longer than three nucleotides. Furthermore, a significant sequence identified by FIMO found within the *TTHA0653/4* promoter contains a central G/C-rich region with a four nucleotide C-tract (Fig. S6). However, this sequence showed no significant protection compared with a control sequence by REPA (Fig. 3, *C* and *D*). These data argue that TTHA1953 has a unique DNA sequence recognition compared with previously studied CsoR–RcnR members (6, 20).

Through *in vitro* and *in vivo* experimentation, we provide strong evidence that TTHA1953 represses transcription units beginning with the following genes: *TTHA1730*, *TTHA1422*, *TTHA1326*, and *TTHA1411*. *TTHA1730* encodes an uncharacterized protein that is predicted to be membrane bound, while the remaining transcription units encode proteins involved in sulfur metabolism, whether through the Sox pathway or through a separate sulfite oxidation pathway (*TTHA1326*). Since the Sox pathway takes place in the periplasmic space, it is enticing to imagine membrane-bound TTHA1730 as a downstream player in this pathway; however, more experimentation is needed to address this hypothesis. We also show that *TTHA1423*, the divergent gene upstream of *TTHA1422*, is also controlled by TTHA1953. Similar to *TTHA1326*, *TTHA1423* encodes for a cytochrome *c*-552 precursor; however, the biological role of TTHA1423 is currently unknown.

### Relationship between TTHA1953 and CstR

TTHA1953 shares several similarities to the CstR. Both proteins appear to be modulated through cysteine reactivity and repress genes involved in sulfur metabolism. However, there are some major differences between TTHA1953 and experimentally validated CstR homologs. Notably, all established CstR proteins are found in organisms that do not contain a putative Sox system. In contrast, CstR homologs often repress rhodaneses, sulfite exporters, and/or sulfide:quinone oxidoreductases (30), which may be used in the defense against hydrogen sulfide stress rather than as a source of energy production. These repressed genes are often found in close proximity to *CstR* (30), whereas the transcription regulatory network of TTHA1953 is vastly separated from the genomic *TTHA1953* position. CstR homologs have also been shown to sense reactive sulfur species, often promoting intermolecular disulfide bonding and subsequent reduction in DNA-binding affinity (30, 31, 32). Although we show that disulfide-linked TTHA1953 has a lower DNA-binding affinity (Fig. 2, *B*–*E*), we did not observe a reduction in TTHA1953 DNA-binding when incubated with increasing amounts of the reactive sulfur species, NaHS (Fig. S10). Nonetheless, we cannot exclude the possibility that other reactive sulfur species may influence DNA binding of TTHA1953. Interestingly, we observed a growth delay in *TTHA1953*-mutant *T. thermophilus* HB8 cultures grown in rich media, whereas other *CstR*-mutant bacteria only show growth defects when cultured with reactive sulfur species (31). Taken together, we believe that TTHA1953 most closely aligns with the CstR subfamily based on functional studies; however, the differences discussed previously should be noted.

### A novel player of Sox regulation

Although *Sox* operons encode for critical machinery in SOB, their genetic regulation has not been extensively characterized. To date, few transcriptional regulatory mechanisms have been established. One is the two-component system found in acidophilic, SOB (*Acidithiobacillus*). The two-component system contains TspS and TspR, where TspS senses sulfide stress and TspR activates *Sox* expression (33). A similar two-component system may be used in the purple sulfur bacterium *Allochromatium vinosum*, although the implicated regulators have no homology to TspR/S (34).

Another level of transcription regulation is through SoxR, which may also be in a two-component system with SoxS (not to be confused with the superoxide response regulator genes [SoxR/S] with the same nomenclature). These proteins have been identified in several alphaproteabacteria, including *Paracoccus pantotrophus* and *Pseudaminobacter salicylatoxidans* (35, 36). SoxR is a member of the ArsR family of transcriptional regulators (37) and was found to bind multiple intergenic sequences between Sox genes. SoxR likely functions as a transcriptional repressor to the Sox pathway; however, conflicting results were observed at different SoxR expression levels (36).

In this study, we provide the first evidence of *Sox* regulation by a CsoR-like protein in the model extremophile *T. thermophilus* HB8. Natural habitats of *T. thermophilus* often contain high levels of sulfur cycling (38), and we show that dysregulation of sulfur metabolism genes directly hinders *T. thermophilus* HB8 growth. Our data suggest that the transcription regulation of TTHA1953 can be modulated by disulfide bond formation, but the mechanism of how this covalent linkage occurs *in vivo* is yet to be determined. Continued characterization of proteins within the TTHA1953 transcription regulatory network may help determine the molecular switch that governs the DNA-binding affinity of TTHA1953. Collectively, this study adds the *Sox* operon to the regulatory repertoire of the CsoR–RcnR family of transcription factors and highlights the apparent diversity in *Sox* regulation.

## Experimental procedures

### Oligonucleotides

All oligonucleotides used in this study were purchased from Integrated DNA Technologies and are presented in File S1. Oligonucleotides used for REPSA, REPA, BLI, or EMSA were amplified using ST2L and ST2R primers. Where indicated, the ST2R primer was conjugated with 5′ IRDye-700 or 5′ biotin. In REPA reactions, the control DNA template was amplified using ST2R and 5′ IRDye-800-labeled ControlL primers. Unless otherwise stated, DNA templates were amplified by PCR using New England Biolabs (NEB) Taq DNA polymerase with standard Taq buffer under reaction conditions specified by the manufacturer.

### Expression and purification of TTHA1719 and TTHA1953

Expression vectors containing the TTHA1719 (TEx02C07) and TTHA1953 (TEx04A02) sequences were purchased from RIKEN BioResource Research Center (39). An expression vector for TTHA1953_C39A C68A_ was created using gene synthesis cloning through GenScript. Briefly, the coding sequence of *TTHA1953* was modified to mutate each of the two cysteine residues to alanine. The resulting DNA sequence went through codon optimization for *Escherichia coli* expression and then was cloned and inserted into a pET-11a backbone using NdeI and BamHI restriction sites. *E. coli* strain Rosetta 2 (DE3)-competent cells were transformed with each expression vector and grown on ampicillin-containing agar. Ampicillin-resistant clones were then isolated, grown in 50 ml LB media at 37 °C and 250 rpm to an absorbance of 0.5, induced with 1 mM IPTG, then allowed to continue growth for an additional 4 h. Each culture was pelleted and resuspended in 1 ml 2× extraction buffer (40 mM Tris–Cl [pH 7.5], 200 mM NaCl, 0.2 mM EDTA, 2 mM DTT, and 1 mM PMSF). To lyse the cells, resuspended pellets were treated with ∼250 μg lysozyme for 10 min on ice, then sonicated at 3 W, 10 s on/50 s off, for five cycles. Sonicated mixtures were pelleted by centrifugation. For TTHA1719 purification, the soluble fraction after sonication was heated to 70 °C for 15 min to denature endogenous *E. coli* proteins and then centrifuged at 4 °C, 14,000*g* for 15 min. The resulting supernatant was collected, diluted twofold with 100% glycerol, and stored at −20 °C. For TTHA1953 and TTHA1953_C39A C68A_ purification, the pellet after sonication was washed once with 2× extraction buffer containing 1 M NaCl to extract chromatin-bound proteins. After centrifugation, the supernatant was heated to 70 °C for 15 min to denature endogenous *E. coli* proteins and then centrifuged at 4 °C, 14,000*g* for 15 min. The resulting supernatant was collected, diluted twofold with 100% glycerol, and stored at −20 °C. All protein samples were found to be over 95% pure by SDS-PAGE and Coomassie staining.

### REPSA

REPSA was performed as described in detail previously (17). Briefly, 2 ng of a IRDye-700 labeled PCR-amplified selection template library (oligo “STR24” in File S1) was incubated with 60 nM TTHA1953 in a 20 μl reaction containing 1× CutSmart buffer (NEB) and 1 mM DTT. We also set up two control reactions lacking the purified transcription factor. Reactions were placed at 55 °C for 20 min and then transferred to 37 °C for 5 min. The TTHA1953-containing reaction, as well as one control reaction, was treated with 0.4 units FokI (NEB), incubated at 37 °C for 5 min, and then placed on ice to stop FokI cleavage. Samples from each reaction were diluted in 1× orange gel loading dye (NEB). The TTHA1953-containing reaction was then aliquoted and subjected to 6, 9, or 12 cycles of PCR amplification. Samples from each PCR were diluted in 1× orange load dye, and all DNA samples were then analyzed by 10% native PAGE and visualized using a LI-COR Odyssey imager. The PCR containing the most dsDNA template was purified using a DNA Clean and Concentrator-5 kit (Zymo Research). Purified PCR product was quantified using a Qubit 3 Fluorometer (ThermoFisher). This purified DNA was then incubated with 60 nM TTHA1953, and the aforementioned process was repeated until a protected DNA species was observed by gel electrophoresis (round 6, Fig. 1*A*). To avoid IISRE-specific DNA sequences (40), each round of REPSA alternated between using 0.4 units of FokI or BpmI (NEB), as indicated.

### REPA

REPAs were performed in 10 μl reactions containing 1× CutSmart buffer, 5 nM experimental DNAs labeled with IRDye-700, 5 nM control DNAs labeled with IRDye-800, and the indicated concentration of TTHA1953. Unless otherwise noted, reactions contained 1 mM DTT. Each REPA experiment contained two control reactions: one that lacks both TTHA1953 and IISRE and one that lacks TTHA1953. These controls serve as molecular weight markers for uncut and cut DNAs, respectively. Reactions were incubated at 55 °C for 20 min and then transferred to 37 °C for 5 min. Reactions were then treated with 0.2 units FokI for 5 min at 37 °C. Reactions were quenched with the addition of 2.2 μl 6× orange gel loading dye supplemented with 1% SDS. Samples were then separated by 10% native PAGE and visualized using a LI-COR Odyssey imager. Where indicated, reactions were supplemented with 100 μM CuCl_2_, 100 μM H_2_O_2_, 100 μM NiCl_2_, 100 μM CoCl_2_, or increasing amounts of NaHS.

### DNA sequencing and bioinformatics

DNAs from round 6 of REPSA with TTHA1953 were sequenced using an Illumina iSeq100 system following the manufacturer’s instructions. Sequencing libraries were prepared by fusion-cloning REPSA-selected DNAs using appropriate barcoded primers. Sequencing data were output as individual fastq files, and a library of 24-nucleotide long sequences was created by processing sequencing reads with our Sequencing1.java script to remove flanking regions. To identify consensus sequence motifs, refined sequencing libraries were inputted into MEME, version 5.4.1 using default parameters. Position weighted matrices of the most significant consensus motif were then mapped to the *T. thermophilus* HB8 genome using FIMO, version 5.4.1 using default parameters. Sequences that fell within −200/+20 nucleotides of a translation start site were identified using the Kyoto Encyclopedia of Genes and Genomes *T. thermophilus* HB8 database (40) (https://www.genome.jp/kegg-bin/show_organism?org=T00220). Predicted functional roles of *T. thermophilus* HB8 gene products were identified using GenBank (41), and operons were identified using predicted regulatory elements from Softberry BPROM and FindTerm (42).

### BLI

BLI was performed as described in detail previously (43). Briefly, 2 ng of DNA template was subjected to 20 rounds of PCR amplification using a 5′biotin-labeled primer, allowing for conjugation to streptavidin-coated Dip and Read biosensors (FortéBio). The buffer used throughout each BLI experiment was 20 mM Tris (pH 7.5), 100 mM NaCl, 1 mM EDTA, 0.05% Tween-20, and 1 mM DTT. Each experiment was performed at 37 °C and included the following steps: 100 s start up, 900 s loading, 100 s baseline, 500 s association, and 500 s dissociation. Shift values corresponding with the association and dissociation steps were initially normalized to the shift value observed immediately prior to association. Then, these values were input into GraphPad Prism 9 (GraphPad) to create the graphs presented in Figure 1*D*. *K*_*d*_ values were predicted using the nonlinear regression, *Association then Dissociation* model.

### Design of TTHA1953-disruption plasmid

Regions upstream and downstream of *TTHA1953* were amplified from the *T. thermophilus* HB8 genome (isolated using the Quick-DNA Fungal/Bacterial Miniprep kit [Zymo Research]) using the 1953_UpF_HindIII/1953_UpR_KpnI and 1953_DownF_PstI/1953_DownR_AatII primers (File S1), respectively. The thermostable kanamycin resistance (*KanR*) gene, as well as its accompanying promoter, was amplified from the *TTHA1292*-disruption plasmid (RIKEN Bioresource Research Center, TDs07G02) using ProKan_F_KpnI/ProKan_R_PstI primers (File S1). The downstream region of *TTHA1953* and the *KanR* fragment was digested with PstI (NEB) and ligated with T4 ligase (NEB). The resulting product was amplified using the ProKan_F_KpnI/1953_DownR_AatII primers and digested with KpnI (NEB). The upstream region of *TTHA1953* was also digested with KpnI and ligated to the *TTHA1953*Downstream/*KanR* fusion product. This fragment was isolated by gel extraction (Zymoclean Gel DNA Recovery kit [Zymo Research]), digested with HindIII (NEB) and AatII (NEB), and ligated into a pUC19 backbone. MAX Efficiency Stbl2-competent cells (ThermoFisher) were transformed with the resulting ligation reaction, and successful transformations were isolated on kanamycin-containing agar plates. Ultimately, this created a plasmid containing ∼500 bp of genomic sequences found upstream or downstream of *TTHA1953*, separated by the *KanR* sequence.

### Creating TTHA1953-mutant *T. thermophilus* HB8

The reference strain of *T. thermophilus* HB8 (American Type Culture Collection 27634) was cultured in TT media (0.8% polypeptone peptone, 0.4% yeast extract, 0.2% NaCl, 0.4 mM MgCl_2_, and 0.4 mM CaCl_2_) overnight at 70 °C, shaking at 180 RPM. The overnight culture was diluted 500-fold into 500 μl TT media and grown for 60 min at 70 °C and 180 RPM. The culture was then supplemented with 2 μg of *TTHA1953*-disruption plasmid and continued growth for 6 h. About 200 μl of the culture was plated on TT plates (0.8% polypeptone peptone, 0.4% yeast extract, 0.2% NaCl, 1.5 mM MgCl_2_, 1.5 mM CaCl_2_, and 1.5% gellan gum) containing 500 μg/ml kanamycin. Three kanamycin-resistance colonies were cultured, and genomic DNA was isolated using a Quick-DNA Fungal/Bacterial Miniprep kit (Zymo Research). Disruption of the *TTHA1953* gene was verified by genomic PCR using the qPCR_1953_F/qPCR_1953_R primers as well as primers for the 16s rRNA gene, PCR_16S_F/PCR_16S_R, as a positive control (File S1 and Fig. S7).

### *T. thermophilus* HB8 growth, RNA isolation, and qPCR

All reference and *TTHA1953*-disrupted strains were streaked onto TT plates prior to growth in liquid media. Single colonies from each streak were inoculated in TT media and grown at 70 °C, 180 RPM. For growth curves, starter cultures were grown overnight, and equivalent cell counts were inoculated into 30 ml cultures. Absorbance at 600 nm readings was performed by Synergy H1 Hybrid Reader (BioTek). For gene expression analysis, 1 ml samples of reference or *TTHA1953*-disrupted *T. thermophilus* HB8 cultures were collected and pelleted. RNA was isolated using a Quick-RNA Fungal/Bacterial Miniprep kit (Zymo Research). Resulting nucleic acid was treated with five units of DNaseI (Zymo Research) for 15 min at room temperature and then purified using an RNA Clean & Concentrator kit (Zymo Research). Complementary DNA (cDNA) libraries were generated using First-Strand cDNA synthesis kit (APExBIO) following the manufacturer’s protocol. Gene expression was quantified by qPCR using the qPCR primers listed in File S1. qPCRs were assembled in a 10 μl volume containing 1× concentrate of iTaq Universal SYBR Green Supermix (Bio-Rad), 500 nM of each forward and reverse primer (File S1), and 2 μl diluted cDNA library. Expression levels were normalized to the endogenous pyruvate dehydrogenase E1 component gene (*TTHA0185*).

### *In vitro* transcription assay

Promoter templates were designed to include sequences ∼200 nts upstream and ∼200 nts downstream of the indicated translation start site. Primers used to amplify the *TTHA1422/3*, *TTHA1411*, and *23S* promoter templates from *T. thermophilus* HB8 genomic DNA are presented in File S1. Promoter templates were purified by a DNA Clean & Concentrator kit (Zymo Research). *In vitro* transcription assays were performed in 1× CutSmart buffer supplemented with 1 mM DTT, and all reactions were incubated at 65 °C. About 10 ng/μl promoter template was preincubated with 5 μM TTHA1953 or a buffer control for 5 min. Reactions were then supplemented with 100 μM *T thermophilus* HB8 RNA polymerase core and sigma factor (rpoD), which were purified as described previously (44), and incubated for 5 min. To start the reaction, NTPs were added to 0.3 mM, and reaction samples were taken at 0, 10, and 20 min after NTP addition. Samples were immediately treated with 0.5 units DNaseI for 10 min at room temperature, then diluted with TBE–urea buffer (40 mM Tris–Cl [pH 8.3], 45 mM boric acid, 1 mM EDTA, 8 M urea, 6% Ficoll-400, and 0.01% bromophenol blue). Samples were heated at 65 °C for 2 min, separated by 5% TBE–urea PAGE, and then visualized with SYBR Gold staining (Molecular Probes).

## Data availability

All data described are contained within the article with the exception of raw DNA sequencing files, which can be provided upon request.

## Supporting information

This article contains supporting information.

## Conflict of interest

The authors declare that they have no conflicts of interest with the contents of this article.
