# Supplementary material for: A CsoR family transcriptional regulator, TTHA1953, controls the sulfur oxidation pathway in Thermus thermophilus HB8
Source: J Biol Chem. 2023 Apr 26;299(6):104759. doi: 10.1016/j.jbc.2023.104759 (PMC10318464; doi:10.1016/j.jbc.2023.104759)
Supplement: Figures S1–S10 [file mmc2.pdf]

Supplemental Information for:

A CsoR family transcriptional regulator, TTHA1953, controls the sulfur oxidation pathway in *Thermus thermophilus* HB8

John K. Barrows and Michael W. Van Dyke#

Department of Chemistry and Biochemistry, Kennesaw State University, Kennesaw, Georgia, USA

#Address correspondence to Michael W. Van Dyke, mvandyk2@kennesaw.edu.

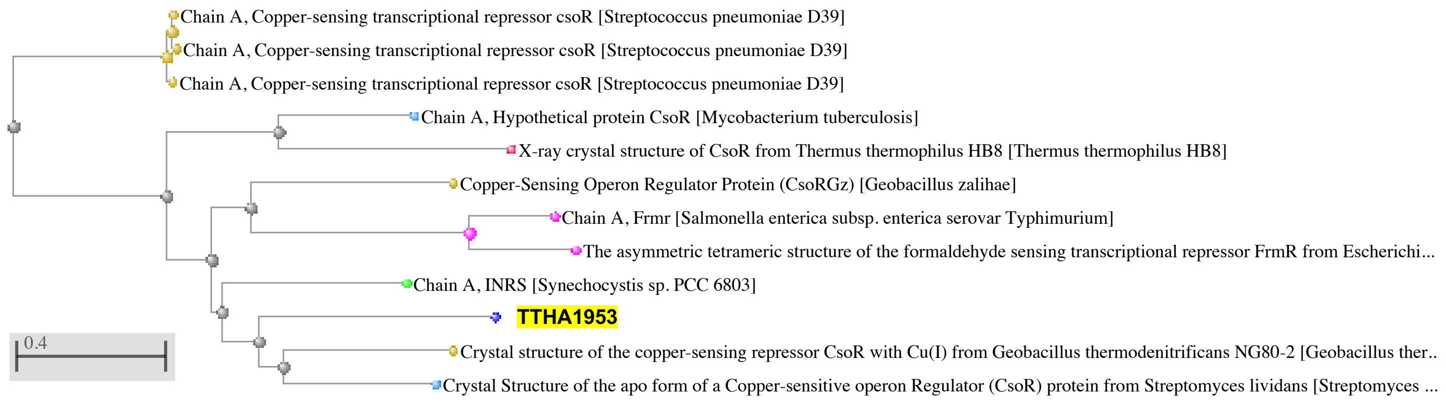

Figure S1: PSI-Blast of TTHA1953. Distance tree analysis from three iterations of PSI-BLAST using the TTHA1953 amino acid sequence. Analysis was restricted to the Protein Data Bank database.

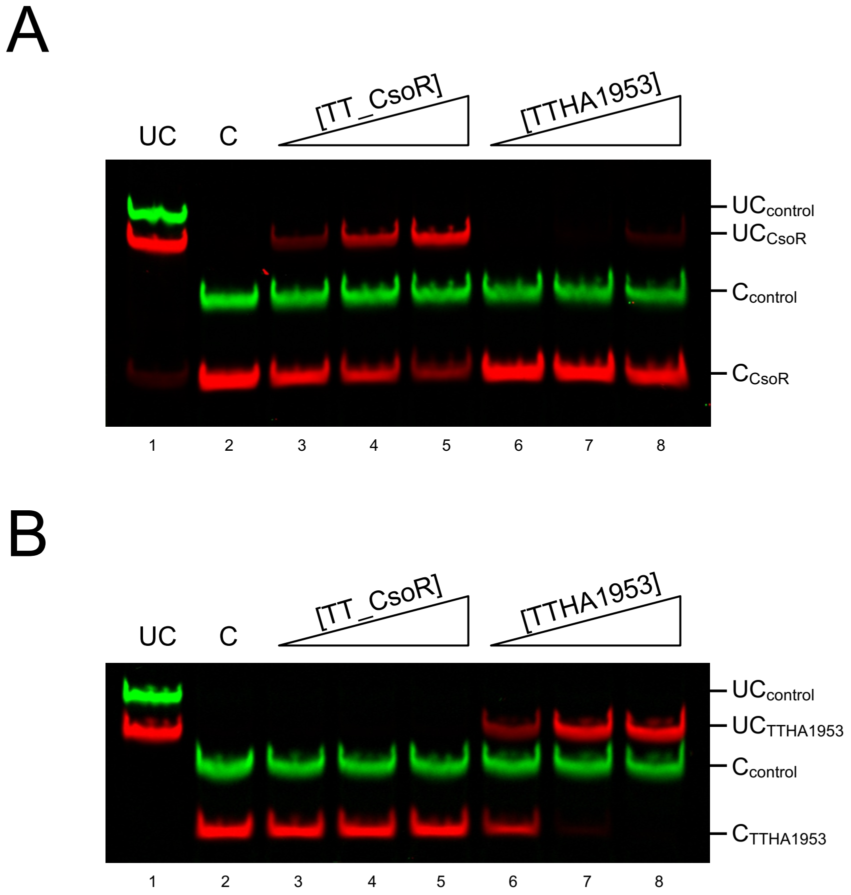

Figure S2: *Thermus thermophilus* HB8 CsoR homologs, TTHA1953 and TTHA1719, bind unique DNA sequences. (**A**) DNAs containing the predicted TTHA1719 (TT_CsoR) DNA-binding sequence (5' IRDye700-labeled; red) and control DNAs with no sequence homology (5'IRDye800-labeled; green) were incubated with 30, 60, or 120 nM TT_CsoR or TTHA1953, then treated with FokI. Uncut (UC) and cut (C) DNAs are labeled. (**B**) DNAs containing our consensus TTHA1953 DNA-binding sequence (5' IRDye700-labeled; red) and control DNAs with no sequence homology (5'IRDye800-labeled; green) were incubated with 30, 60, or 120 nM TT_CsoR or TTHA1953 for 20 minutes, then treated with FokI. Uncut (UC) and cut (C) DNAs are labeled. To promote TT_CsoR binding, reactions in (**A**) and (**B**) were performed with 3 mM EDTA. Note: the addition of EDTA did not alter the binding ability of TTHA1953.

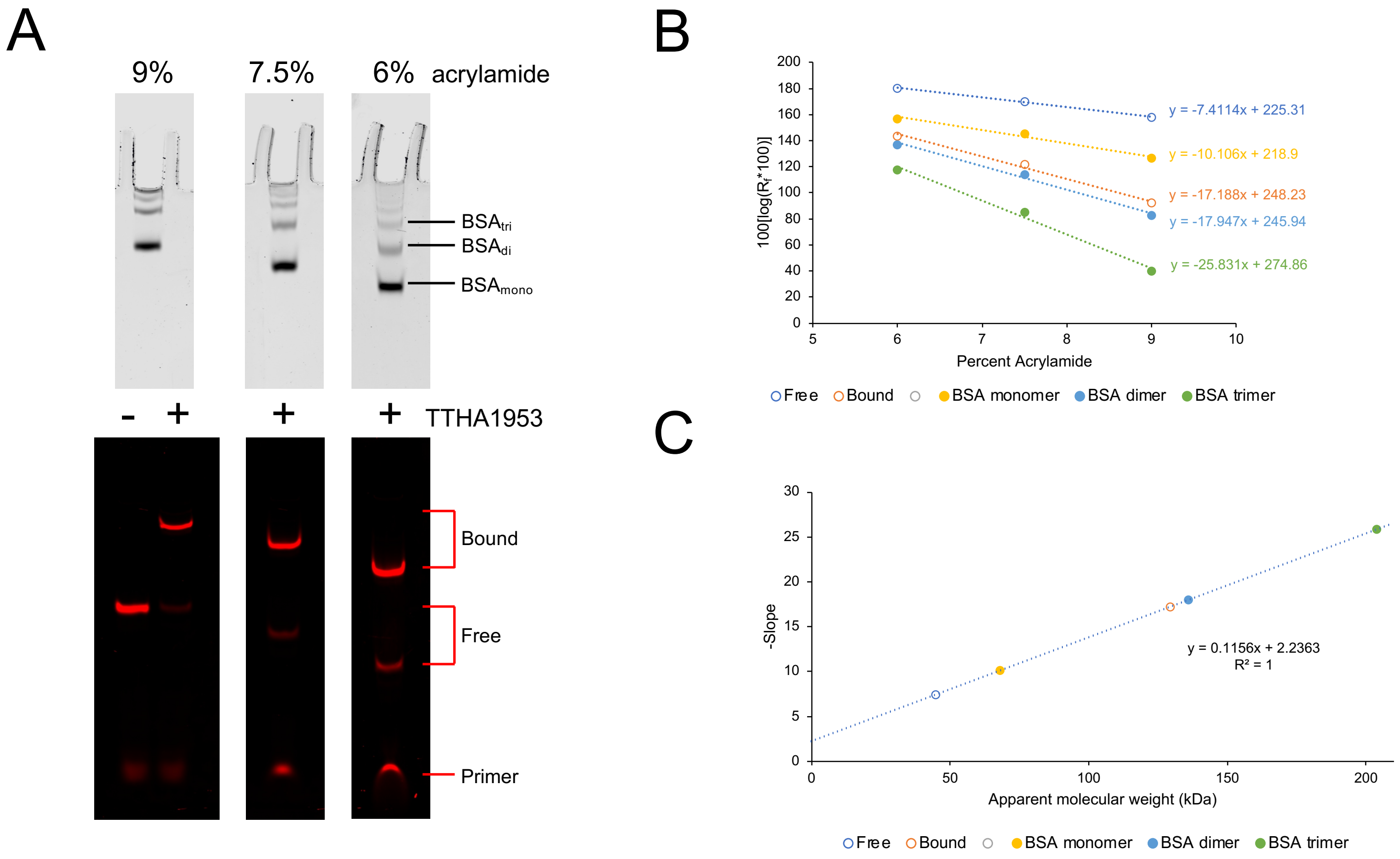

Figure S3: TTHA1953 binds consensus sequence DNA as a homo-octomer complex. (**A**) DNAs containing our consensus TTHA1953 DNA-binding sequence were incubated with 120 nM TTHA1292, separated by native PAGE containing the indicated percentage of acrylamide, and visualized using a LI-COR Odyssey imager (lower panels). Each gel also contained samples of 2 µg BSA that were visualized by Coomassie staining (upper panels). (BSA), position of monomer (mono), dimer (di) and trimer (tri) BSA complexes; (Bound), shifted DNA species consistent with TTHA1953-binding; (Free) unbound DNA species; (Primer), free primers remaining after PCR of the DNA template. (**B**) Relative mobility (R_f) was calculated by determining the ratio of the experimental band migration (denoted in the legend) and the loading dye migration for each sample in each gel condition. Linear regressions of the indicated species are presented. (**C**) Graph of the negative slope from the regression models determined in (**B**) and apparent molecular weight. BSA oligomers, for which molecular weights are known, are shown in closed circles. Open circles represent species from the binding reactions in (**A**). A linear regression model was fit to the BSA samples, and the equation and R^2 value is shown. The apparent weight for the unknown species in (**A**) was identified using the presented linear regression model: Free, 44.7 kDa; Bound, 129 kDa.

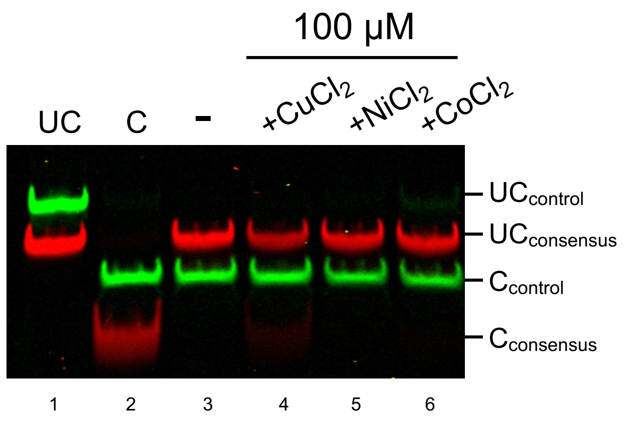

Figure S4: Metal ions do not abolish TTHA1953 DNA-binding. DNAs containing our consensus TTHA1953 DNA-binding sequence (5’ IRDye700-labeled; red) and control DNAs with no sequence homology (5’IRDye800-labeled; green) were incubated with 100 nM TTHA1953. Reactions contained 100 µM of the indicated metal. After a 20-minute incubation, reactions were treated with FokI. Uncut (UC) and cut (C) DNAs are labeled.

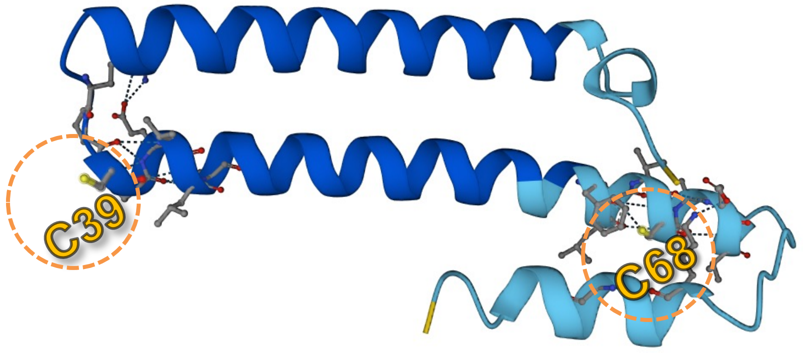

Figure S5: Predicted structure of TTHA1953 by AlphaFold. The predicted ribbon structure of TTHA1953 is shown. The two cysteine residues (C39 and C68) are labeled. A mutant TTHA1953 construct containing alanine substitutions for both cysteine residues (TTHA1953$_{C39A\ C68A}$) is used in **Figure 2E**.

A

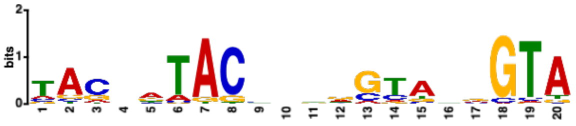

C

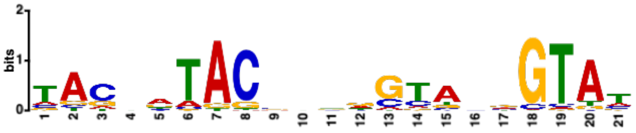

B

Chromosome	Start	End	Strand	Distance to TSS (bp)	Gene	p-value	Q-value	Matched Sequence
1	1622624	1622643	+	-17<br>-58	*TTHA1730*<br>*TTHA1729*	3.60E-08	0.149	AACAATACCGGGCTAGGGTA
1	1351633	1351652	+	-150<br>+14	*TTHA1423*<br>*TTHA1422*	1.19E-06	1	TACGGTACACCGCCAGGGTA
1	1265658	1265677	+	-22	*TTHA1326*	2.27E-06	1	CCCTATACCCAGCTAGGGTA
1	612828	612847	+	N/A	N/A	2.87E-06	1	AAAGCTACGAGAGCAACGTA
1	1143167	1143186	-	-6	*TTHA1198*	3.06E-06	1	CTTCTTACCTTGCTAAAGTA
1	1143162	1143181	+	-1	*TTHA1198*	3.16E-06	1	ACCCCTACTTTAGCAAGGTA
1	891506	891525	-	-84	*TTHA0943*	3.32E-06	1	CACAACACCAAGCTTTGGTA
pTT27	118364	118383	-	-4	*TTHB128*	3.43E-06	1	AACGAAACAAAAGGGAGGTA
1	614917	614936	-	N/A	N/A	3.72E-06	1	TACCGTAAACTTGAAGAGTA
1	1471552	1471571	-	-23	*TTHA1543*	3.78E-06	1	AAGTAAGCTTAGCTATGGTA
pTT27	143824	143843	+	143	*TTHB153*	4.16E-06	1	TCGCAAAGGGAAGTAAAGTA
1	1622624	1622643	-	-17<br>-58	*TTHA1730*<br>*TTHA1731*	4.58E-06	1	TACCCTAGCCCGGTATTGTT
1	49464	49483	-	-6<br>+7	*TTHA0050*<br>*TTHA0049*	4.65E-06	1	TAGAATACGGGGGTGGAGAA
1	1182724	1182743	+	N/A	N/A	4.80E-06	1	TAGTAGACGTAGGTCATGTA
1	574529	574548	-	+3<br>-65	*TTHA0607*<br>*TTHA0606*	4.95E-06	1	TCGTAAACCAAAATAAACTA
1	612455	612474	-	-116	*TTHA0643*	5.62E-06	1	TAATTTACATCGGTGGTCTA
1	612833	612852	-	N/A	N/A	6.17E-06	1	CACTCTACGTTGCTCTCGTA
1	472986	473005	-	-41	*TTHA0508*	6.57E-06	1	CACTATACCTCGCCTGTGTA
1	621270	621289	-	-11<br>-44	*TTHA0654*<br>*TTHA0653*	6.57E-06	1	GAGTATACCCCAGGATAGTT
1	1507328	1507347	+	-100	*TTHA1584*	6.67E-06	1	TAAGATACAAGAGTATGTCA
1	587635	587654	-	+5<br>-29	*TTHA0618*<br>*TTHA0617*	8.27E-06	1	CACCATATCCCGCTAGTGTA
1	890907	890926	-	N/A	N/A	8.40E-06	1	TAGATAACCTTTTTATTGTG
pTT27	109461	109480	-	N/A	N/A	8.53E-06	1	TGCTTTGCGAAGCTTTAGTA
1	614917	614936	+	N/A	N/A	8.93E-06	1	TACTCTTCAAGTTTACGGTA

D

Chromosome	Start	End	Strand	Distance to TSS (bp)	Gene	p-value	Q-value	Matched Sequence
1	1622624	1622644	+	-16<br>-58	*TTHA1730*<br>*TTHA1729*	7.40E-09	0.0313	AACAATACCGGGCTAGGGTAT
1	621269	621289	-	-11<br>-43	*TTHA0654*<br>*TTHA0653*	1.75E-06	1	GAGTATACCCCAGGATAGTTT
1	1351633	1351653	+	-149<br>+13	*TTHA1423*<br>*TTHA1422*	2.51E-06	1	TACGGTACACCGCCAGGGTAC
1	584274	584294	-	+5	*TTHA0615*	2.76E-06	1	GACCATACCAATCCTTAGTTT
1	1143162	1143182	+	-1	*TTHA1198*	2.80E-06	1	ACCCCTACTTTAGCAAGGTAA
1	1507327	1507347	-	-100	*TTHA1584*	2.85E-06	1	TGACATACTCTTGTATCTTAT
pTT27	118363	118383	-	-3	*TTHB128*	3.03E-06	1	AACGAAACAAAAGGGAGGTAA
1	136829	136849	-	-30<br>-68	*TTHA0140*<br>*TTHA0139*	3.18E-06	1	CATCATACTCAAGTTGAGCAT
pTT27	213472	213492	+	-6	*TTHB204*	3.18E-06	1	TAGTATACCAAAAAGTCGTGT
1	1343244	1343264	+	+3	*TTHA1411*	3.18E-06	1	CCATATACCCTCCTAACGTAT
1	1507323	1507343	+	-104	*TTHA1584*	3.28E-06	1	TCCGATAAGATACAAGAGTAT
1	1622628	1622648	-	-12<br>-62	*TTHA1730*<br>*TTHA1729*	3.28E-06	1	ACCTATACCCTAGCCCGGTAT
1	1471551	1471571	-	N/A	N/A	3.33E-06	1	AAGTAAGCTTAGCTATGGTAA
1	584270	584290	+	+1	*TTHA0615*	3.90E-06	1	CGCTAAACTAAGGATTGGTAT
1	1622623	1622643	-	-17<br>-57	*TTHA1730*<br>*TTHA1729*	4.02E-06	1	TACCCTAGCCCGGTATTGTTA
1	574528	574548	-	+3<br>-64	*TTHA0607*<br>*TTHA0606*	4.35E-06	1	TCGTAAACCAAAATAAACTAA
1	358435	358455	+	-13	*TTHA0375*	4.42E-06	1	TGCTATACTCCGGCAGGGAAT
1	1265658	1265678	+	-21	*TTHA1326*	4.55E-06	1	CCCTATACCCAGCTAGGGTAC
1	1270182	1270202	+	+1	*TTHA1330*	4.55E-06	1	TAGCGTTCGGAGGTGAGGTAT
1	1343248	1343268	-	-1	*TTHA1411*	4.77E-06	1	GGGCATACGTTAGGAGGGTAT
1	1816980	1817000	+	+1<br>-43	*TTHA1937*<br>*TTHA1936*	5.47E-06	1	TCGGCTACAATGGGAGGGTAT
1	109928	109948	-	-30	*TTHA0109*	5.73E-06	1	TTCACTAAGCTAGTAAGGTTT
1	1507328	1507348	+	-99	*TTHA1584*	5.82E-06	1	TAAGATACAAGAGTATGTCAA
1	1059225	1059245	+	-48<br>-45	*TTHA1112*<br>*TTHA1111*	6.97E-06	1	AAGCCTACCTATGTCAAGTTT
1	1471547	1471567	+	-18	*TTHA1543*	8.34E-06	1	TATGTTACCATAGCTAAGCTT
1	612828	612848	+	N/A	N/A	8.47E-06	1	AAAGCTACGAGAGCAACGTAG
1	1143166	1143186	-	-5	*TTHA1198*	8.99E-06	1	CTTCTTACCTTGCTAAAGTAG
1	1571366	1571386	-	N/A	N/A	9.53E-06	1	ACCAAAACAACAATACGGTTT
1	891505	891525	-	-83	*TTHA0943*	9.67E-06	1	CACAACACCAAGCTTTGGTAG

Figure S6: Position weight matrices inserted into FIMO for gene templates depicted in Figure 3. (**A**) A variation of the position weighted matrix identified in **Figure 1B**. (**B**) The position weighted matrix in (**A**) was inserted into FIMO and searched against the *Thermus thermophilus* HB8 genome. Significant genomic locations (p > 10E-5) are presented. Distance to TSS, distance in base pairs (bp) to the nearest translation start site; Gene, gene name corresponding to the indicated translation start site; N/A, the genomic sequence was not found within -200/+20 bps of a translation start site. (**C**) A variation of the position weighted matrix identified in **Figure 1B**. (**D**) The position weighted matrix in (**C**) was inserted into FIMO and searched against the *Thermus thermophilus* HB8 genome. Significant genomic locations (p > 10E-5) are presented. Distance to TSS, distance in base pairs (bp) to the nearest translation start site; Gene, gene name corresponding to the indicated translation start site; N/A, the genomic sequence was not found within -200/+20 bps of a translation start site.

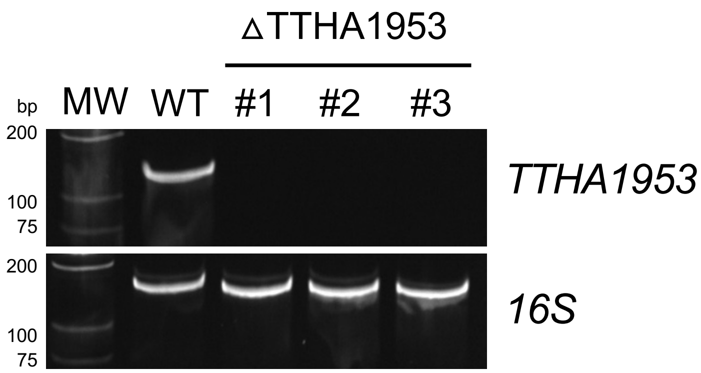

Figure S7: Genomic PCR to validate TTHA1953 mutants. Genomic DNA was isolated from three kanamycin-resistance clones of *Thermus thermophilus* HB8 following incubation with a *TTHA1953*-disruption plasmid. DNA samples were subjected to 25 rounds of PCR using primers specific for the *TTHA1953* or *16S* genes (primer sequences are in File S1). PCR products were analyzed by 10% native PAGE and visualized by ethidium bromide staining. (MW), molecular weight marker; (WT), wild-type/reference *Thermus thermophilus* HB8 genomic DNA; (bp), base pairs.

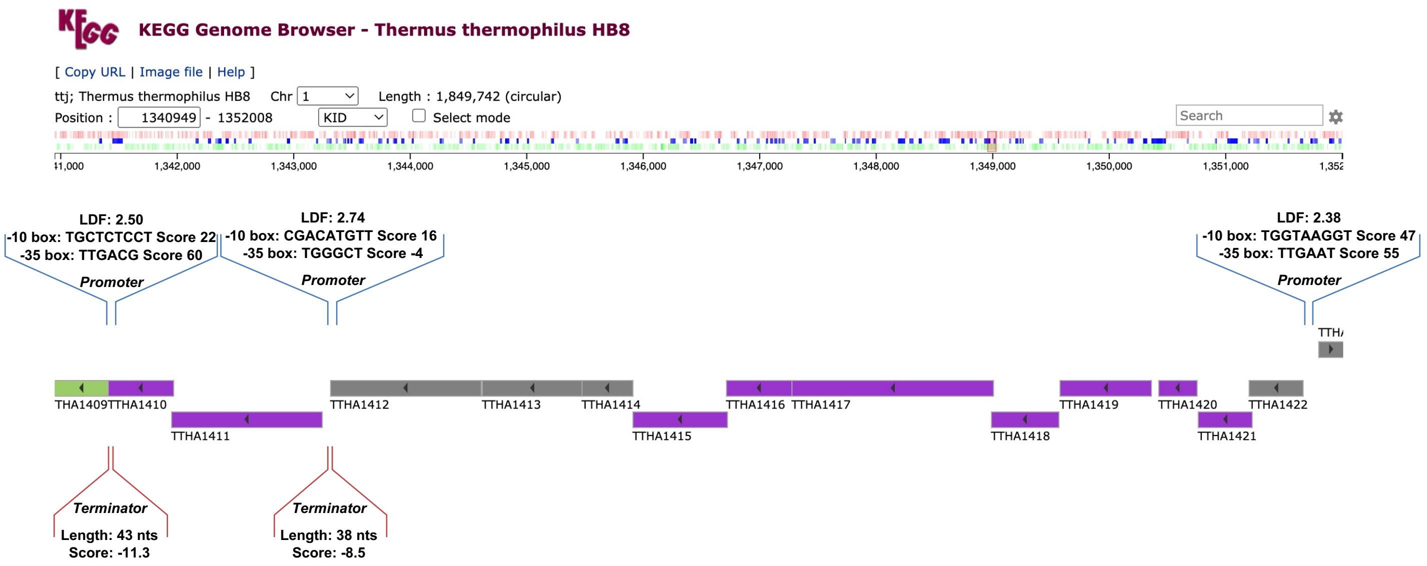

Figure S8: Promoter and terminator sequences identified in *Thermus thermophilus* HB8 *Sox* locus. A screenshot of the KEGG Genome Browser for *Thermus thermophilus* HB8 is presented. Putative promoter and terminator sequences were determined by the Softberry services, BPROM and FindTerm, respectively. The queried sequence spanned the genes *TTHA1422* - *TTHA1410* containing ~200 nts on either side. Shown are the terminator sequences found with a score function < -8 and promoter sequences with an LDF > 2.0. (LDF), linear discriminant function.

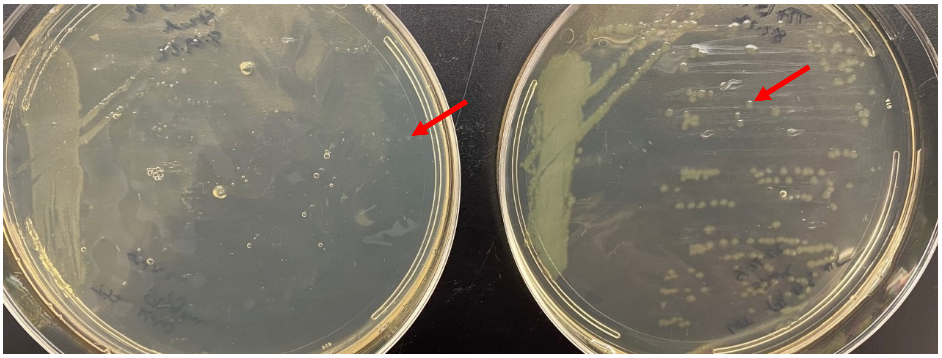

ΔTTHA1953 Reference

Figure S9: Growth of Δ*TTHA1953* and reference strain on solid media. Glycerol stocks of reference and *TTHA1953*-disrupted *Thermus thermophilus* HB8 strains were streaked onto TT plates and incubated at 70°C for 16 hours. Representative single colonies are marked by the red arrow.

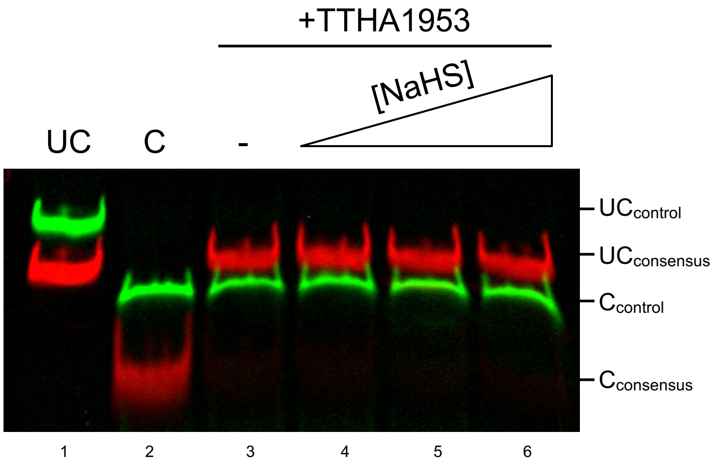

Figure S10: NaHS does not antagonize TTHA1953 DNA binding. DNAs containing our consensus TTHA1953 DNA-binding sequence (5' IRDye700-labeled; red) and control DNAs with no sequence homology (5'IRDye800-labeled; green) were incubated with 100 nM TTHA1953 in the absence of a reducing agent. Where indicated, reactions contained 10, 100, or 1000 μM NaHS. After a 20-minute incubation, reactions were treated with FokI. Uncut (UC) and cut (C) DNAs are labeled.
